# Evaluation of the practicability and virological performance of finger-stick whole-blood HIV self-testing in French-speaking sub-Saharan Africa

**DOI:** 10.1371/journal.pone.0189475

**Published:** 2018-01-10

**Authors:** Serge Tonen-Wolyec, Salomon Batina-Agasa, Jérémie Muwonga, Franck Fwamba N’kulu, Ralph-Sydney Mboumba Bouassa, Laurent Bélec

**Affiliations:** 1 Ecole Doctorale Régionale D’Afrique Centrale en Infectiologie Tropicale, Franceville, Gabon; 2 Faculté de Médecine, Université de Bunia, Bunia, Democratic Republic of the Congo; 3 Faculté de Médecine et de Pharmacie, Université de Kisangani, Kisangani, Democratic Republic of the Congo; 4 Laboratoire National de Référence du Sida, Kinshasa, Democratic Republic of the Congo; 5 Programme National de lutte Contre le VIH/SIDA et les IST, Kinshasa, Democratic Republic of the Congo; 6 Laboratoire de virologie, hôpital Européen Georges Pompidou, and Université Paris Descartes, Paris Sorbonne Cité, Paris, France; University of California, San Francisco, UNITED STATES

## Abstract

**Background:**

Opportunities for HIV testing could be enhanced by offering HIV self-testing (HIVST) in populations that fear stigma and discrimination when accessing conventional HIV counselling and testing in health care facilities. Field experience with HIVST has not yet been reported in French-speaking African countries.

**Methods:**

The practicability of HIVST was assessed using the prototype the Exacto^®^ Test HIV (Biosynex, Strasbourg, France) self-test in 322 adults living in Kisangani and Bunia, Democratic Republic of the Congo, according to World Health Organization’s recommendations. Simplified and easy-to-read leaflet was translated in French, Lingala and Swahili.

**Results:**

Forty-nine percent of participants read the instructions for use in French, while 17.1% and 33.9% read the instructions in Lingala and Swahili, respectively. The instructions for use were correctly understood in 79.5% of cases. The majority (98.4%) correctly performed the HIV self-test; however, 20.8% asked for oral assistance. Most of the participants (95.3%) found that performing the self-test was easy, while 4.7% found it difficult. Overall, the results were correctly interpreted in 90.2% of cases. Among the positive, negative, and invalid self-tests, misinterpretation occurred in 6.5%, 11.2%, and 16.0% of cases, respectively (P<0.0001). The Cohen’s κ coefficient was 0.84. The main obstacle for HIVST was educational level, with execution and interpretation difficulties occurring among poorly educated people. The Exacto^®^ Test HIV self-test showed 100.0% (95% CI; 98.8–100.0) sensitivity and 99.2% (95% CI; 97.5–99.8) specificity.

**Conclusions:**

Our field observations demonstrate: (i) the need to adapt the instructions for use to the Congolese general public, including adding educational pictograms as well as instructions for use in the local vernacular language(s); (ii) frequent difficulties understanding the instructions for use in addition to frequent misinterpretation of test results; and (iii) the generally good practicability of the HIV self-test despite some limitations. Supervised use of HIVST is recommended among poorly-educated people.

## Introduction

Reaching universal HIV status awareness is crucial to ensure that all HIV-infected patients have access to antiretroviral treatment and achieve virological suppression. Diagnosing 90% of all people with HIV is the first of three global 90–90–90 targets set by the United Nations to end the HIV epidemic by 2030 [1]. The first target of the UNAIDS initiative, however, remains a key challenge [2]. Indeed, despite achievements in scaling up HIV testing during the last 10 years, more than 45% of people living with HIV in sub-Saharan Africa are unaware of their HIV status [3]. Pitfalls of HIV testing in health care facilities include fears over loss of privacy and confidentiality, as well as potential stigma and discrimination, especially in young people and marginalized high-risk groups, such as female sex workers and homosexual men [4–6].

HIV self-testing (HIVST) represents an innovative strategy to expand access to HIV testing services in the general population and also to reach individuals at high risk for HIV who may not otherwise submit to HIV testing, including young people and key populations [7,8]. In 2012, oral HIVST was approved by the US Food and Drug Administration [9]. In 2015, HIVST was authorized in Europe [10]. In low-income countries, regulated self-test kits are generally not yet available for the general public [6]. However, recent pilot studies conducted in Kenya [11,12], Malawi [13], Nigeria [14], Uganda [15] and South Africa [16,17] have demonstrated high acceptability and uptake of HIVST in sub-Saharan Africa [18]; these field studies have also shown that interventions involving HIVST may be effective in linking self-testers to effective HIV care. Based on these preliminary observations, HIVST has been proposed as a way to address the challenges of efficiently reaching people at risk for or with an undiagnosed HIV infection who may not otherwise receive testing [19]. The World Health Organization (WHO) encourages countries to pilot and explore how HIVST can be used to scale up HIV testing, especially among populations not reached by existing HIV testing services [6,20]. Donors and stakeholders are also currently evaluating whether investments should be made to support the development, promotion, and marketing of HIVST in low-income countries [6,21,22]. In December 2016, the WHO formally recommended HIVST as an additional approach to HIV testing services [23,24]. Finally, the WHO has published technical recommendations to develop and validate HIV self-tests [25,26].

Pilot programs in the field will provide the experiences needed to both deliver HIVST in an effective, ethical, and acceptable way and to inform country implementation and policy. In Africa, the opportunities and barriers addressed by HIVST have been until now exclusively explored in English-speaking countries [6,27–29]. To our knowledge, the feasibility and practicability of HIVST has never been reported in French-speaking African countries, which have their own specific cultural, economic, and societal characteristics. Finally, the aim of this study was to carry out the practicability evaluation of HIVST in Kisangani and Bunia, Democratic Republic of the Congo (DRC), using a prototype HIV self-test and according to recent WHO recommendations [25].

## Material and methods

### Prototype HIV test for self-testing

The CE IVD, lateral flow, immunochromatographic HIV rapid test [from Biosynex, Strasbourg, France, as *Own Brand Labeller*, under the trademark Exacto^®^ PRO Test HIV] [6,21,22] was adapted as a prototype finger-stick whole-blood HIV self-test (Exacto^®^ Test HIV, Biosynex). The test uses a specific antibody-binding protein that is conjugated to colloidal gold dye particles and synthetic antigens (gp41, gp36) able to detect antibodies against HIV-1 or HIV-2 in whole blood, serum, or plasma, which are bound to the solid phase membrane. The Exacto^®^ Test HIV fulfilled the following criteria: (i) capillary blood-based test detecting early HIV infection with analytical sensitivity in primary HIV infection previously evaluated at 92% [30]; (ii) sterile safety lancet; (iii) simplified blood sampling system; (iv) simplified buffer delivery system; (v) specimen presence control by blood deposit assessment and migration control band; and (vi) results in 10 minutes.

### Study design

The practicability evaluation of the HIV self-test comprised a multicenter cross-sectional study performed between March and June 2016 in Kisangani and Bunia, the capital cities of the DRC provinces of Tshopo and Ituri, respectively, consisting of face-to-face and self-administered questionnaires, according to WHO recommendations [25].

### Study population

Volunteers were included from five voluntary and counselling testing (VCT) sites for HIV infection as well as sites involved in the care of HIV-infected individuals at the health centers of Neema and Boyoma in Kisangani, the *Hôpital Général de Référence de Bunia*, the *Centre hospitalier Bunia cité* and the *Clinique Mpabenda* of Bunia. Recruited volunteers were asked for socio-demographic and personal information, including, sex, age, known ongoing pregnancy, sexual orientation, partnership and civil status, occupation, educational level, number of sex partners in the past six months, past history of HIV counselling and testing, knowledge of own HIV serostatus and antiretroviral treatment.

### Recruitment

All participants were volunteers recruited from the general public (patients, their visitors/relatives) or health care workers (doctors, nurses, laboratory technicians) at the study sites. Inclusion of a limited number of health care workers was recommended by the WHO [25]. These participants, including health care workers, were either people seeking to know their HIV status or people living with HIV. The inclusion criteria were as follows: age ≥ 18 years; self-professed ability to read the instructions for use of the HIV self-test in French, Lingala, or Swahili; willingness to undergo HIV screening; and willingness to give written informed consent to participate in the study. The exclusion criteria were age < 18 years, illiteracy, and unwillingness to follow protocol instructions.

### Laboratory procedures

Blood was drawn by venipuncture in EDTA tubes on site from each participant and centrifuged at 1000 rpm for 15 min; the plasma was then aliquoted and kept frozen at -4°C until use at the Reference Provincial Laboratory of Kisangani.

Serological HIV testing at the five inclusion sites was first carried out according to the national algorithm of the DRC Ministry of Public Health, based on a WHO II algorithm for HIV testing [31]. In brief, 50 μl of whole blood was serologically tested for HIV-1/HIV-2 antibodies in series using Alere Determine^™^ HIV-1/2 (Alere Medical Co. Ltd., Matsudo-shi, Chiba-ken, Japan) as a first test and Uni-Gold^™^ HIV (Trinity Biotech Manufacturing Ltd., Bray, Co. Wicklow, Ireland) was used as a second confirmatory test [31].

All positive and indeterminate samples by initial WHO II algorithm screening were further re-tested on plasma in the Reference Provincial Laboratory of Kisangani with Alere Determine^™^ HIV-1/2 (Alere Medical Co. Ltd.) and Uni-Gold^™^ HIV (Trinity Biotech Manufacturing Ltd.) as the first two HIV tests, followed by a third HIV testing using recomLine HIV-1 and HIV-2^®^ IgG (Biosynex), according to the 2012-revised, 2015-consolidated WHO strategy for HIV testing [19, 32]. HIV serological results were negative when the three HIV tests were negative, positive when they were positive or indeterminate when they gave discordant results.

HIV viral load was assessed in the reference laboratory of Kinshasa by molecular biology using the Abbott Real-Time HIV-1 assay (Abbott Molecular Inc, IL, USA) on plasma from all HIV-seropositive samples, and one out of two HIV-seronegative plasmas was randomly selected [dx.doi.org/10.17504/protocols.io.k34cyqw].

### Design of the instructions for use of HIVST and translation into vernacular languages

**Fig 1:**
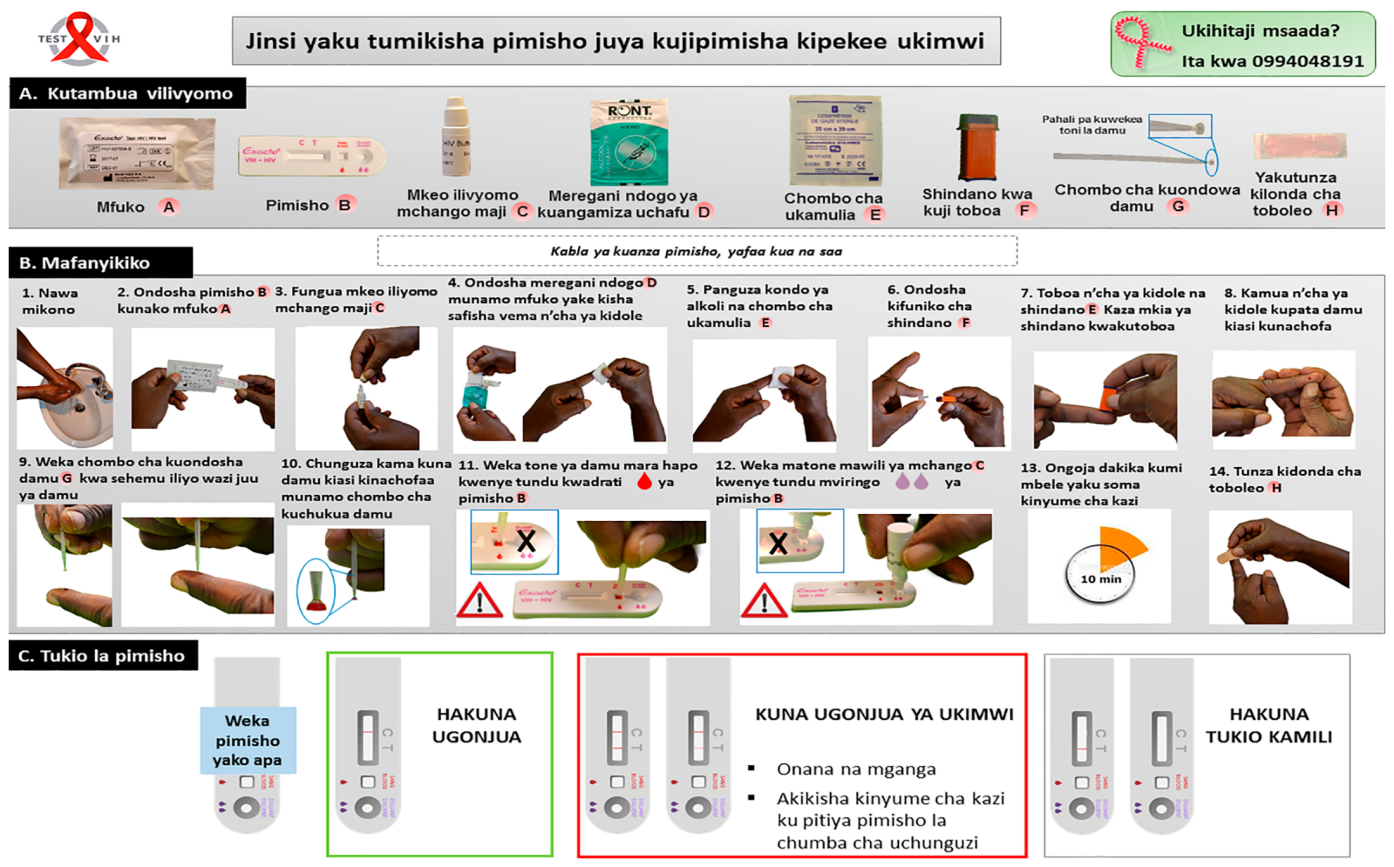
Instructions for use of the Exacto^®^ Test HIV (Biosynex) self-test designed for the Congolese general public using typical pictures representative of the principal steps of the manufacturer’s instructions with explanations written in Swahili, the most frequently used vernacular language of the former Province Orientale of the Democratic Republic of the Congo. Other available languages were French and Lingala. **A. Identification of the components: Ⓐ** Bag, **Ⓑ** Test cassette, **Ⓒ** Diluent vial, **Ⓓ** Disinfectant wipe, **Ⓔ** Compression swab, **Ⓕ** Lancet, **Ⓖ** Sampler stick, **Ⓗ** Dressing. **B. Performing the self-test**: 1. Wash your hands; 2. Take the self-test out of the bag **Ⓐ**; 3. Open the diluent vial **Ⓒ**; 4. Disinfect the chosen fingertip with the disinfectant wipe **Ⓓ**; 5. Wipe off residual alcohol with the compression swab **Ⓔ**; 6. Apply the lancet **Ⓕ** on the chosen fingertip and push the other tip to sting; 7. Press gently on the fingertip to obtain a drop of blood; 8. Place in contact the drop of blood with the sampler stick **Ⓖ** until the inverted cup becomes full; 9. Check that the sampler tip **Ⓖ** is filled with blood; 10. Place the blood into the SQUARE well BLOOD of the test cassette **Ⓑ**; 11. Shed two drops of diluent in the ROUND well DILUENT of the test cassette **Ⓑ**; 12. Wait exactly 10 minutes before reading the result.

The original instructions for use of the Exacto^®^ Test HIV were adapted into a simplified but comprehensive version for the Congolese general public, with illustrations showing African people carrying out the test. The simplified instructions for use in French were further translated into Lingala and Swahili, which together constitute the most widely used vernacular languages in the provinces of Tshopo and Ituri. Ultimately, the simplified instructions for use of the HIV self-test comprised an easy-to-read leaflet in French, Lingala and Swahili, in A3 format color printing. As an example, the instructions for use in Swahili are depicted in Fig 1.

### Practicability study outcomes

The practicability evaluation was divided into three substudies carried out by trained health care professionals according to 2015 WHO recommendations [25]. A structured questionnaire was used to obtain socio-demographic data and evaluate the participants’ understanding of the instructions for use and their opinions or levels of satisfaction about the practicability of the HIV self-test.

#### Substudy 1: Comprehension of labeling

After participants were provided with an explanation of the objectives of the research, they were asked to move to a private room where the instructions for use of the Exacto^®^ Test HIV were given for reading and understanding in their preferred language (French, Lingala or Swahili). After reading the instructions for use, the participants were asked whether they agreed to continue participating in the study by completing a self-administered evaluation questionnaire to gauge their comprehension of the instructions for use. Eight questions restating the key instructions for use with closed answers (true or false) were asked by the observer on the followings items: 1. Identification of each component of the kit; 2. Manipulation of blood sampling device; 3. Diluent deposit; 4. Possession of a timer; 5. Interpretation of a negative test result; 6. Diagnosis of an invalid test result; 7. Practical consequences of a positive test result; 8. Interpretation of a negative test result three weeks after high risk unprotected sexual intercourse.

The participants who correctly answered a minimum of five questions were considered to have correctly understood the instructions, whereas those who correctly answered less than five questions were considered to have not understood the instruction, as previously described [33]. The participants used the same instructions for use for the remainder of the study.

A satisfaction questionnaire concerning the instructions for use allowed for the collection of information concerning the contents of the kit, experiences with the HIV self-test, interpretation of the self-test results, overall understanding of the instruction for use, and their usage in the local language.

#### Substudy 2: Interpretation of the results

All participants from substudy 1 were evaluated concerning the ability to read and interpret the results of the HIV self-test. In a private room supervised by an observer, 13 standardized tests (including three positive, five negative, two invalid and three positive with low test strips) were proposed to the participants for interpretation after successive random selection. These standardized tests were coded by numbers to determine the expected results.

A satisfaction questionnaire concerning the interpretation of HIV self-test results allowed for the collection of information concerning the reading and interpretation of a positive test, a negative test, and an invalid test, as well as the determination of whether the blood deposit in the SQUARE well was correctly observed.

#### Substudy 3: Observation of manipulation

In a confidential room supervised by an observer, each participant received a box containing the Exacto^®^ Test HIV kit. Participants were then asked to carry out HIVST by themselves. The health care professional observer was responsible for recording the respect or not of each step. He was also responsible for recording the appeal for oral assistance (mimicking telephone support), failure factors, and the elapsed time to perform HIVST (since opening the box to the migration stage) on a standardized sheet. The health care observer had received rigorous training, including how to talk to participants asking for any support concerning the HIVST. The successful performance of the HIV self-test was conditioned by the presence of the control strip, and the test results were read and recorded independently by both the participants and the observers. At the end of the session, each participant was asked to fill out a satisfaction questionnaire. Finally, the participants were moved to the next room with trained staff members for blood sampling. Note that the voluntary counselling and testing service provided pre-test and post-test counselling. Participants were told to consider only the results of HIV testing according to the national HIV screening algorithm because the HIV self-test should be used only for research purposes.

A satisfaction questionnaire concerning experiences with the HIV self-test allowed for the collection of information regarding recognition of the components of the HIV self-test, the overall performance of the self-test, and the ability to surmount the difficulties encountered.

### Analytical evaluation of HIVST Exacto^®^ Test HIV

The sensitivity and specificity of the Exacto^®^ Test HIV as read by observers were calculated according to the results of the serologic testing algorithms or the molecular biology for HIV RNA load measurement as reference diagnosis methods for HIV infection. Furthermore, the positive predictive values (PPV) and negative predictive values (NPV) were calculated by taking into account the reported HIV prevalence of 2.3% in the sexually active population of the former Province Orientale of DRC [34] using Bayes’ formulae [35].

### Statistical analysis

All data were entered into an Excel file and analyzed on SPSS 20.0 (Chicago, IL). Descriptive statistics were computed. Means and standard deviations (SD) were calculated for quantitative variables and proportions for categorical variables. The variable “educational level” included three categories: low (unschooled and primary schooled), middle (college or technical school), and high (undergraduate degree and graduate degree) educational levels. The results were presented as a 95% confidence interval (CI) using the Wilson score bounds. The Pearson’s χ_2_ test was used for comparison of the frequencies, while Fisher’s exact test was used when the validity conditions of the latter test were not verified. Comparisons of means used the Student’s *t* test or ANOVA for variables with more than two classes. The concordance between the results read by participants in connection with the expected results or as read by operators was estimated by Cohen’s κ coefficient. The degree of agreement was determined as ranked by Landlis and Koch [36]. The accuracy of the Exacto^®^ Test HIV to correctly diagnose HIV infection was estimated by Youden’s J index (J = sensitivity + specificity − 1) [37]. Finally, to delineate and control possible confounders within the study variables and determine the independent predictors of the understanding of instructions for use (substudy 1), the correct interpretation of the HIV self-test results (substudy 2), the need for oral assistance, and the successful performance of the HIV self-test (substudy 3), multivariable logistic regression analysis was carried out using significant variables from the bivariate analysis, which were arbitrarily taken as references for analyses. Missing data in multivariate logistic regression analysis were assigned the null value for conservative estimates. The strength of statistical associations was measured by crude and adjusted odds ratios (OR) and their 95% confidence intervals. The P-value < 0.05 was considered as statistically significant.

### Ethics statement

The study was conducted after obtaining ethical approval from the Ethics Committee of the School of Public Health of the University of Kinshasa, constituting the National Ethics Committee of the DRC. Furthermore, permission was obtained from provincial divisions of health of the provinces of Tshopo and Ituri of the DRC. Informed consent was obtained from all volunteers in writing. No personal information from the participants was registered to ensure anonymity. Volunteers were also informed that they could withdraw at any time from the study without any consequences. The study was conducted by the Research, Teaching, and Care Unit (URES) of the Faculty of Medicine and Pharmacy of Kisangani University and by the National AIDS Reference Laboratory.

## Results

### Study population

The demographic characteristics and past medical history of the study population are presented in Table 1. A total of 336 volunteers were recruited for the study, but 14 were excluded because they were minors (less than 18 years old) (*n* = 12) or considered noncompliant (*n* = 2). Finally, 322 participants, including 144 (44.7%) from Kisangani and 178 (55.3%) from Bunia, participated in the practicability study. Among them, 297 (92.2%) were members of the general public and 25 (7.8%) were health care workers. Female participants constituted 64.6% of the sample population. The majority were less than 30 years old. All were heterosexual. Around one-half were single or married. One-third were students, one-third were employed, and one-third were unemployed. Low educational level (unschooled and primary school) was observed in 16.5% of participants; a middle level (college or technical school) was observed in 44.1% and a good or high level (bachelor’s degree, graduate degree or postgraduate degree) was observed in 39.4% of participants. The number of sexual partners during the last six months was generally from one to five. More than one-half of participants (54.3%) had yet received HIV counselling and testing in the past, of whom 23.4% (41/175) were followed-up at clinical centers for HIV infection; among those who were HIV positive, 63.4% (26/41) claimed they were taking antiretroviral treatment with cotrimoxazole prophylaxis, and the remaining 36.6% (15/41) were receiving only prophylactic treatment with cotrimoxazole.

**Table 1:** Demographic characteristics and medical history of the 322 study participants.

Variable	Items	Number (%)
**Sex**		
	Male	114 (35.4)
	Female	208 (64.6)
**Age** (**years**)		
	18–29	197 (61.2)
	30–39	80 (24.8)
	≥ 40	45 (14.0)
**Partnership and civil status**		
	Single	147 (45.7)
	Married/partnered	154 (47.8)
	Separated or divorced	16 (5.0)
	Widowed	5 (1.6)
**Occupation**		
	Student	111 (34.5)
	Employed	93 (28.9)
	Unemployed	118 (36.6)
**Educational level** *		
	Low	53 (16.5)
	Middle	142 (44.1)
	High	127 (39.5)
**Number of sex partners in the past six months**		
	0	50 (15.5)
	1–5	262 (81.4)
	6–10	7 (2.2)
	> 10	3 (0.9)
**Previous HIV counselling and testing**		
	Yes	175 (54.3)
	No	147 (45.7)
**Previously diagnosed HIV positive among those previously HIV tested** ^μ^		
	Yes	41 (23.4)
	No	134 (76.6)
**HIV care after knowledge of HIV infection**^#^		
	Antiretroviral therapy and cotrimoxazole prophylaxis	26 (63.4)
	Cotrimoxazole prophylaxis only	15 (36.6)

* Educational level was categorized according to the educational system of the Democratic Republic of the Congo, as follows: (i) low: unschooled or attending primary school; (ii) middle: attending college (training of six years) or technical school (training of four years); and (iii) high: attending Bachelor’s degree or graduate degree (training of two years after Bachelor’s degree); none of the study participants were attending a postgraduate degree;

^**μ**^ Overall, 53 participants were finally found positive, 41 of which previously knew their positive HIV-status. Concerning the basic positivity found in this study, 12 of 276 participants who needed a first or new testing were diagnosed as HIV positive, giving a prevalence of 4.3% of HIV infection;

^#^ All participants were taken cotrimoxazole prophylaxis, including 26 of them on antiretroviral treatment.

### Practicability evaluation

The practicability evaluation included the following three substudies, according to WHO recommendations [25].

#### Substudy 1

Substudy 1 evaluated the ability of the 322 study participants to understand the instructions for use of the Exacto^®^ Test HIV. The instructions for use in vernacular languages (Lingala and Swahili) were used more often (50.9%) than those written in French (49.1%).

All health care workers (*n* = 25; 7.8%) had used the French instructions for use. Among the general public (*n* = 297; 92.2%), the instructions for use in French, Lingala, and Swahili were used by 44.8% (*n* = 133), 18.5% (*n* = 55), and 36.7% (*n* = 109) of participants, respectively (data not shown). All participants had taken sufficient time to read the instructions for use of the Exacto^®^ Test HIV. The analytical results of the evaluation questionnaire are shown in Table 2A. Overall, 256 (79.6%; 95% CI: 74.7–83.6) participants correctly understood the instructions for use, thus correctly answering at least five questions (Table 2B). Most (94.1%) participants correctly responded to the issue concerning the identification of kit components. By contrast, a minority (40.4%) of participants correctly responded to the issue related to a negative self-test result three weeks after high-risk of sexual intercourse.

**Table 2:** Analytical results of the evaluation questionnaire (A) and number of correct answers to the eight issues raised by question type (B) concerning the ability of the 322 study participants to understand the instructions for use of the Exacto^®^ Test HIV (Biosynex). The questions raising specific issues concerning the manipulation of the kit, the interpretation of test results, and the consequences of the test results were asked by the observer and the answers were closed.

A. Analytical results of the evaluation questionnaire			B. Answers to issues raised by question type		
Issue raised by question type	Answers	Number (%)	Number of correct answers*	Number (%)	[95% CI]
Q1: “A capital letter is associated with each component of the kit to better identify it during the performance of self-test”	True^#^	303 (94.1)	0	3 (0.9)	[0.3–2.7]
	False	19 (5.9)			
Q2: “The blood collection device helps to collect the blood and place it immediately into the SQUARE well of self-test”	True^#^	278 (86.3)	2	11 (3.4)	[1.9–6.0]
	False	44 (13.7)			
Q3: “Two drops of diluent should be placed in the same well as the drop of blood”	True	157 (48.8)	3	23 (7.1)	[4.9–10.5]
	False^#^	165 (51.2)			
Q4: “A timer (watch or mobile) to clock 10 minutes before reading the result is need”	True^#^	282 (87.6)	4	29 (9.0)	[6.4–12.7]
	False	40 (5.9)			
Q5: “Lack of band by test results is interpreted as a negative test”	True	112 (34.8)	5	54 (16.8)	[13.3–21.5]
	False^#^	210 (65.2)			
Q6: “Lack of control band by test results should be interpreted as an invalid test”	True^#^	252 (78.3)	6	54 (16.8)	[13.3–21.5]
	False	70 (21.7)			
Q7: “In case of positive self-test results, a doctor should be consulted to confirm the result”	True^#^	268 (83.2)	7	92 (28.6)	[24.3–34.1]
	False	268 (83.2)			
Q8: “A negative test result three weeks after high-risk, unprotected sexual intercourse signifies lack of contamination”	True	192 (59.6)	8	56 (17.4)	[13.9–22.1]
	False^#^	130 (40.4)			

^#^ Correct answers;

* No one participant answered correctly to only one question;

Q: Question.

The health care workers tended to better understand the instructions for use than the general public, but the difference was not statistically significant (92.0% *versus* 78.5%; *P* > 0.05 [data not shown]). Comprehension of the instructions for use was associated with educational level and language use, whereas instructions for use in vernacular languages were preferentially used by 94.3% (20.7% in Lingala and 73.6% in Swahili) of participants with low educational levels, 68.4% (26.1% in Lingala and 42.3% in Swahili) with middle educational levels, and only 12.6% (4.7% in Lingala and 7.9% in Swahili) with high educational levels (*P* < 0.0001).

Thus, correct interpretation involved only 73.6% of participants with low educational levels and 71.8% with middle educational levels while it was as high as 90.6% for those with high educational levels (*P* < 0.001). Furthermore, correct interpretation involved only 62.5% of participants using the instructions in Lingala, 73.4% of those using the instructions in Swahili and 89.8% of those using the instructions in French (*P* < 0.0001).

The age of participants was associated with comprehension of the instructions for use. Thus, 79.7% (*n* = 197) of participants between 18 to 29 years correctly interpreted the instructions for use, as did 87.5% (*n* = 80) and 64.4% (*n* = 45) of those between 30 to 39 years and 40 years or more, respectively (*P* < 0.01).

In bivariate analysis, the variables “instructions for use understanding,” “age,” “educational level,” and “language used for the instructions for use” were significantly associated.

In multivariate logistic regression analysis, only the variable “language used for instructions for use” remained associated with the “instructions for use understanding—yes/no” (*see* Table 3): The correct understanding of instructions for use was more frequent when it was in French than in Lingala (89.8% *versus* 62.5%, *P* < 0.001; OR: 0.29 [95% CI: 0.15–0.54], adjusted OR: 0.31 [95% CI: 0.12–0.81]) or in Swahili (89.8% *versus* 73.4%, *P* < 0.01; OR: 0.68 [95% CI: 0.39–1.19], adjusted OR: 0.35 [95% CI: 0.14–0.92]).

**Table 3:** Bivariate and multivariate regression analysis of factors associated with the understanding of the instructions for use among the 322 study participants.

Characteristic	Understanding of the instructions of use^£^					
	*n* (%)	Total	cOR [95% CI]	P*	aOR [95% CI]	P**
**Age** (years)						
18–29	157 (79.7)	197	1.03 [0.59–1.79]	< 0.01	Reference	-
30–39	70 (87.5)	80	2.43 [1.14–5.17]	< 0.01	2.73 [1.07–7.00]	0.056
≥ 40	29 (64.4)	45	0.35 [0.18–0.70]	< 0.01	0.66 [0.26–1.69]	0.291
**Educational level**^μ^						
Low	39 (73.6)	53	0.67 [0.34–1.32]	< 0.0001	0.41 [0.12–1.46]	0.170
Middle	102 (71.8)	142	0.43 [0.25–0.75]	< 0.0001	0.46 [0.18–1.20]	0.111
High	115 (90.6)	127	3.67 [1.87–7.19]	< 0.0001	Reference	-
**Language used**						
French	142 (89.9)	158	3.59 [1.96–6.57]	< 0.0001	Reference	-
Lingala	34 (61.8)	55	0.29 [0.15–0.54]	< 0.0001	0.31 [0.12–0.81]	0.017
Swahili	80 (73.4)	109	0.68 [0.39–1.19]	< 0.0001	0.35 [0.14–0.92]	0.034

^£^ The correct answers to a minimum of five questions were considered to have understood the instructions for use;

* P-value calculated using Pearson’s χ_2_ test or Fisher’s exact test;

** P-value calculated using logistic regression analysis;

^μ^ Educational level was categorized according to the educational system of the Democratic Republic of the Congo, as follows: (i) low: unschooled or attending primary school; (ii) middle: attending college or technical school; and (iii) high: attending Bachelor’s or graduate degree.

aOR: adjusted odds ratios; cOR: crude odds ratios; CI: confidence interval; *P*: *P*-value.

Results of the satisfaction questionnaire concerning the understanding of the instructions for use are shown in S1 Table. Most (65.5%) participants found that the instructions for use were very easy to understand, and 31.1% thought they were rather easy; whereas 2.2% found them rather difficult to understand and 1.2% found them very difficult. A large majority (85.4%) of participants had a favorable opinion of the possibility to use the instructions for use in vernacular languages; 49.4% of participants found the use of vernacular language useful, while 36% found it essential.

#### Substudy 2

**Fig 2:**
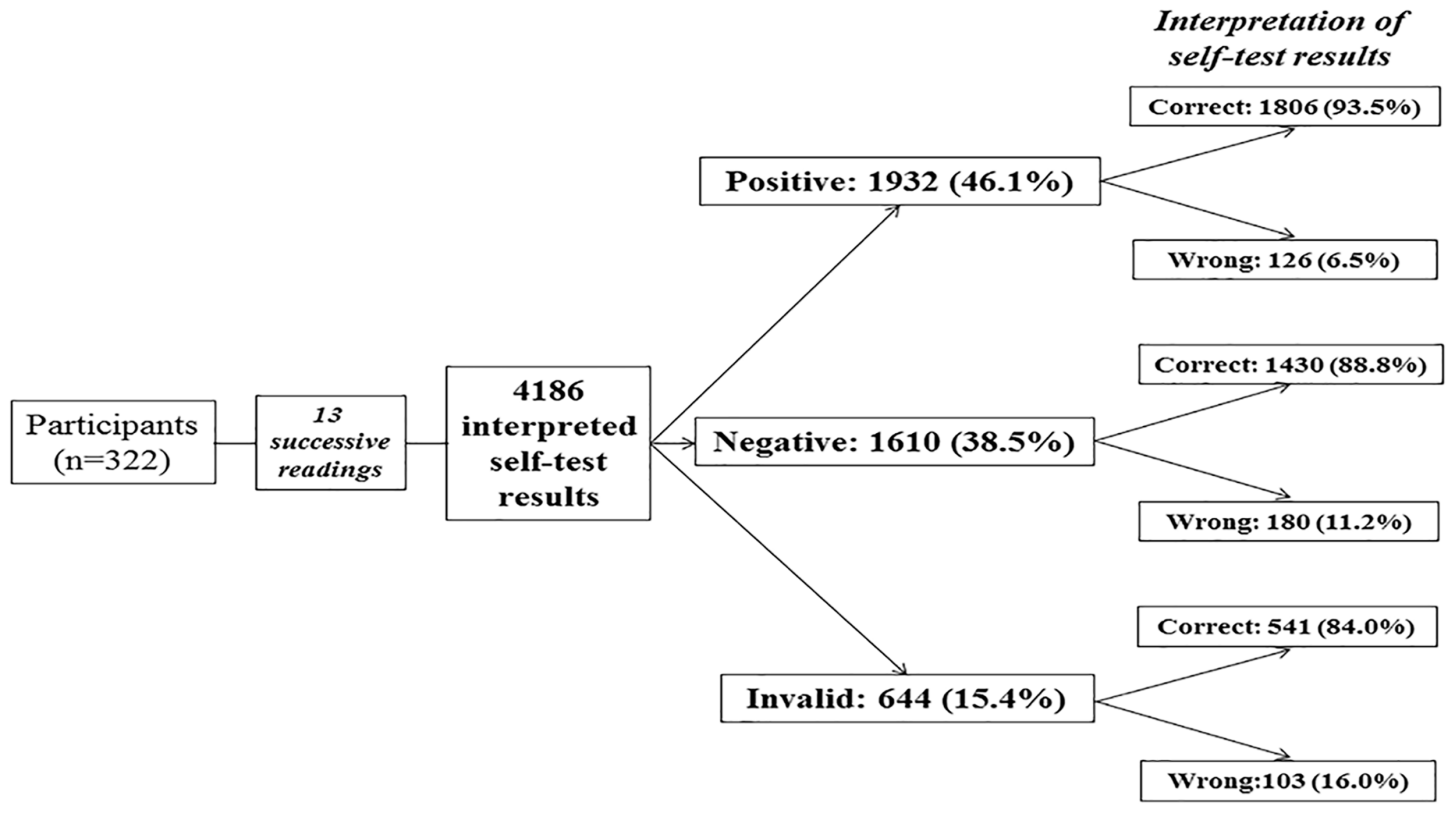
Flow chart showing the ability of participants to read and interpret (correctly or incorrectly) the 4,186 results of the Exacto^®^ Test HIV (Biosynex) obtained from successive random selection of a panel of 13 standardized tests, including six positive, four negative, and two invalid.

Substudy 2 evaluated the ability of participants to read and interpret the HIV self-test results from a panel of 13 standardized tests drawn successively. The results are depicted in Fig 2. A total of 4,186 standardized tests (including 966 positive tests, 1,610 negative tests, 644 invalid tests and 966 positive tests with low test bands) were read and interpreted by the 322 participants; 3,777 (90.2%; 95% CI: 89.3–91.1) tests were correctly interpreted, whereas 409 (9.8%; 95% CI: 8.9–10.7) tests were misinterpreted. Misinterpretation occurred in 16.0% of invalid tests (including 8.9% of tests falsely interpreted as positive and 7.1% as negative); in 11.2% of negative tests (including 8.6% incorrectly interpreted as positive and 2.6% as invalid); and in only 6.5% of positive tests (including 4.4% incorrectly interpreted as negative and 2.1% as invalid). The Cohen’s κ coefficient between the results of reading by participants and the expected results was 0.84, demonstrating excellent concordance according the Landlis and Koch’s rank.

In bivariate analysis, the variables “educational level,” “language used for the instructions for use,” and “understanding of the instructions for use” were factors associated with the interpretation of HIV self-test results. In multivariate logistic regression analysis, the variable “educational level” remained associated with the interpretation of positive and invalid test results (*see* Table 4): Correct interpretation of positive tests was higher in participants with a high educational level than in those with middle (100.0% *versus* 95.1%, *P* < 0.0001; OR: 0.6 [95% CI: 0.2–0.8], adjusted OR: 0.01 [95% CI: 0.00–0.02]) and low (100.0% *versus* 90.6%, *P* < 0.0001; OR: 0.3 [95% CI: 0.1–0.8], adjusted OR: 0.01 [95% CI: 0.00–0.02]) educational levels; correct interpretation of invalid tests was also higher in participants with a high educational level than in those with low (90.6% *versus* 84.9%, *P* = 0.003; OR: 0.4 [95% CI: 0.2–0.7], adjusted OR: 0.5 [95% CI: 0.2–0.8]) educational levels.

**Table 4:** Bivariate and multivariate regression analysis of factors associated with correct interpretation of positive, negative and invalid results among the 4,186 interpreted self-test results by 322 participants.

Characteristic	Correct interpretation of positive self-test						Correct interpretation of negative self-test						Correct interpretation of invalid self-test					
	*n* (%)	Total (N = 1932)	cOR [95% CI]	P*	aOR [95% CI]	P**	*n* (%)	Total (N = 1610)	cOR [95% CI]	P*	aOR [95% CI]	P**	*n* (%)	Total (N = 644)	cOR [95% CI]	P*	aOR [95% CI]	P**
**Educational level**^μ^																		
Low	234 (73.6)	318	0.3 [0.1–0.8]	< 0.0001	0.01 [0.00–0.02]	< 0.0001	220 (83.0)	265	0.5 [0.2–1.2]	< 0.0001	0.6 [0.1–1.2]	0.083	90 (84.9)	106	0.4 [0.2–0.7]	0.006	0.5 [0.2–0.8]	0.003
Middle	810 (95.1)	852	0.6 [0.2–0.8]	< 0.0001	0.01 [0.00–0.02]	< 0.0001	590 (83.1)	710	0.4 [0.2–0.7]	< 0.0001	0.4 [0.1–1.1]	0.063	221 (77.8)	284	1.2 [0.5–2.6]	0.006	0.9 [0.4–2.4]	0.257
High	762 (100)	762	NA	< 0.0001	Reference		620 (97.6)	635	8.4 [2.5–28.1]	< 0.0001	Reference		230 (90.6)	254	2.6 [1.3–5.2]	0.006	Reference	
**Language used**																		
French	888 (93.7)	948	11.4 [1.5–89.6]	0.002	Reference		760 (96.2)	790	3.9 [1.7–8.9]	< 0.0001	Reference		288 (91.1)	316	2.7 [1.4–5.1]	< 0.001	Reference	
Lingala	294 (89.1)	330	0.2 [0.06–0.6]	0.002	0.4 [0.1–1.9]	0.231	205 (74.5)	275	0.3 [0.1–0.7]	< 0.0001	1.3 [0.4–3.8]	0.654	71 (64.5)	110	0.2 [0.1–0.5]	< 0.001	0.4 [0.2–1.0]	0.063
Swahili	624 (95.4)	654	0.7 [0.2–2.3]	0.002	1.2 [0.1–14.9]	0.817	465 (85.3)	545	0.6 [0.3–1.2]	< 0.0001	1.3 [0.4–4.2]	0.654	182 (83.5)	218	1.0 [0.6–1.9]	< 0.001	1.3 [0.5–3.6]	0.614
**Understanding of instructions for use**^£^																		
Yes	1500 (97.7)	1536	4.2 [1.2–13.4]	0.017	2.5 [0.7–8.9]	0.171	1180 (92.2)	1280	3.7 [1.8–7.8]	< 0.0001	1.8 [0.8–3.9]	0.097	454 (88.7)	512	4.8 [2.5–8.9]	< 0.001	1.8 [0.9–3.8]	0.103
No	306 (77.3)	396	Reference	-	Reference		250 (75.8)	330	Reference	-	Reference		87 (65.9)	132	Reference	-	Reference	

* P-value calculated using Pearson’s χ_2_ test or Fisher’s exact test;

** P-value calculated using regression analysis;

^μ^ Educational level was categorized according to the educational system of the Democratic Republic of the Congo, as follows: (i) low: unschooled or attending primary school; (ii) middle: attending college or technical school; and (iii) high: attending Bachelor’s or graduate degree;

^£^ The correct answers to a minimum of five questions were considered to have understood the instructions for use.

OR: adjusted odds ratios; cOR: crude odds ratios; CI: confidence interval; N: Total size; NA: not applicable; *P*: *P*-value.

Results of the satisfaction questionnaire concerning the interpretation of HIV self-test results are shown in S1 Table. Most (93.8%) participants found that the interpretation of positive tests was easy, while 59.3% found it very easy and 34.5% found it rather easy; whereas 6.2% participants responded that it was difficult (including 2.5% who found it rather difficult and 3.7% who found it very difficult).

#### Substudy 3

Substudy 3 evaluated the ability of participants to use the finger-stick whole-blood self-test and get a valid result in a supervised setting. The results of the questionnaire are shown in Table 5.

**Table 5:** Analytical results of the manipulation observation concerning the ability of the 322 study participants to correctly use each step of the Exacto^®^ Test HIV (Biosynex) manipulation autonomously or with oral assistance.

Issue raised by question type	Items	Number (%)
Q1: “The participant recognized the different components of the kit”	Yes	319 (99.1)
	No	3 (0.9)
	Oral assistance	3 (0.9)
Q2: “The participant washed his hands”	Yes	311 (96.4)
	No	11 (3.6)
	Oral assistance	0 (0.0)
Q3: “The participant found the self-test in the bag”	Yes	322 (100.0)
	No	0 (0.0)
	Oral assistance	0 (0.0)
Q4: “The participant opened the diluent vial”	Yes	322 (100.0)
	No	0 (0.0)
	Oral assistance	2 (0.6)
Q5: “The participant disinfected his chosen fingertip with the disinfectant wipe”	Yes	316 (98.1)
	No	6 (1.9)
	Oral assistance	1 (0.3)
Q6: “The participant wiped residual alcohol with the compression swab”	Yes	288 (89.4)
	No	34 (10.6)
	Oral assistance	2 (0.6)
Q7: “The participant applied the lancet on the chosen fingertip and pushed the other tip to sting”	Yes	317 (98.4)
	No	5 (1.6)
	Oral assistance	43 (13.3)
Q8: “The participant pressed gently on the fingertip to obtain a drop of blood”	Yes	309 (96.0)
	No	13 (4.0)
	Oral assistance	2 (0.6)
Q9: “The participant placed in contact the drop of blood with the sampler tip until the tip was full”	Yes	317 (98.4)
	No	5 (1.6)
	Oral assistance	10 (3.1)
Q10: “The participant checked that the sampler tip was filled with blood”	Yes	304 (94.4)
	No	18 (5.6)
	Oral assistance	3 (0.9)
Q11: “The participant placed the blood into the SQUARE well BLOOD of the test cassette”	Yes	317 (98.4)
	No	5 (1.6)
	Oral assistance	13 (4.0)
Q12: “The participant shed two drops of diluent in the ROUND well DILUENT of the test cassette”	Yes	316 (98.1)
	No	6 (1.9)
	Oral assistance	3 (0.9)
Q13: “The participant waited exactly 10 minutes before reading the result”	Yes	302 (93.8)
	No	20 (6.2)
	Oral assistance	0 (0.0)

Q: Question.

Generally, 317 (98.4%; 95% CI: 96.3–99.3) participants correctly used the self-test and succeeded in obtaining an interpretable result, whereas only five (1.6%; 95% CI: 0.7–3.7) participants failed. The correct use of the HIV self-test was observed in 98.3% of the general public and 100% of health care workers (*P* > 0.99 [data not shown]). A total of 255 (79.2%; 95% CI: 75.4–84.2) participants carried out the self-test autonomously, whereas 67 (20.8%; 95% CI: 16.7–25.6) had asked for oral assistance, without a difference between the general public and health care workers (21.2% *versus* 16%; *P* = 0.054 [data not shown]).

Most (64.2%) participants appealing for oral assistance did so when using the lancet. Consequently, the use of a lancet established the failure factor of the HIV self-test performance in 100% of cases (data not shown).

Only 13.2% of participants using the instructions for use in French received oral assistance, as compared to those using the instructions for use in Lingala (31.5%) or Swahili (26.6%) (*P* = 0.003). The level of education influenced the need of oral assistance: 34% of participants with a low level of education asked for oral assistance, while 21.8% with a medium level and 14.2% with a high level (*P* = 0.01) did so. By contrast, the level of education did not influence the correct performance of the HIVST.

The mean time of HIV self-test performance (since the opening of the box until the migration step) was 18±9 minutes. The participants with a low level of education performed the HIV self-test in 21±10 minutes, while those with a middle level performed it in 19±9 minutes and those with a higher level did it in 15±8 minutes (*P* < 0.0001). This difference was also observed in the general public (18±9 minutes) compared to health care workers (11± 7 minutes; *P* < 0.0001 [data not shown]).

All participants (100%) with positive HIV self-tests (*n* = 55) read and interpreted their results correctly. However, four (1.5%) participants with negative HIV self-test results (*n* = 262) interpreted it incorrectly as positive. The Cohen’s κ coefficient of the concordance between the readings by participants and operators was 0.96. Forty-two (76.4%; 95% CI: 63.3–86.0) participants who tested positive knew that the positive HIV self-test needs to be confirmed in a reference laboratory (*P* < 0.05 [data not shown]). Finally, the vast majority of participants correctly recognized the blood deposit in the SQUARE well and the diluent deposit in the ROUND well of the Exacto^®^ Test HIV, in 98.4% and 98.1% of cases, respectively (*see* Table 5).

In bivariate analysis, the variables “successfully performing of the HIVST”, “age”, “educational level”, “language used for the instructions for use”, “understanding of the instructions for use” and “need for oral assistance” were significantly associated.

In multivariate logistic regression analysis, only the variable “need for oral assistance” remained associated only with the “understanding of the instructions for use” (*see* Table 6): The need for oral assistance was more frequent when the instructions for use were poorly understood (37.9% *versus* 16.4%, *P* < 0.001; OR: 0.32 [95% CI: 0.18–0.59]; adjusted OR: 0.44 [0.21–0.91]). Note that correct performing of the HIVST was not associated with any variables.

**Table 6:** Bivariate and multivariate regression analysis of factors associated with the successful performance of the self-test and the need for oral assistance among the 322 study participants.

Characteristic	Need for oral assistance						Successful performance					
	*n* (%)	Total	cOR [95% CI]	P*	aOR [95% CI]	P**	*n* (%)	Total	cOR [95% CI]	P*	aOR [95% CI]	P**
**Age** (years)												
18–29	40 (20.3)	197	0.93 [0.53–1.60]	0.704	Reference	-	192 (97.5)	197	NA	0.186	NA	NA
30–39	19 (23.8)	80	1.14 [0.62–2.11]	0.704	1.51 [0.54–4.16]	0.431	80 (100)	80	NA	0.186	NA	NA
≥ 40	8 (17.8)	45	0.94 [0.43–2.07]	0.704	1.46 [0.55–3.86]	0.446	40 (100)	45	NA	0.186	NA	NA
**Educational level**^μ^												
Low	18 (34.0)	53	2.31 [1.21–4.41]	0.011	1.89 [0.64–5.61]	0.255	52 (98.1)	53	0.79 [0.09–7.17]	0.172	NA	NA
Middle	31 (21.8)	142	1.12 [0.65–1.92]	0.011	0.91 [0.38–2.18]	0.826	138 (97.2)	142	0.19 [0.02–1.74]	0.172	NA	NA
High	18 (14.2)	127	0.49 [0.27–0.89]	0.011	Reference	-	127 (100)	127	NA	0.172	NA	NA
**Language used**												
French	21 (13.2)	158	0.39 [0.22–0.69]	0.003	Reference		158 (100)	158	NA	0.057	NA	NA
Lingala	17 (31.5)	55	2.49 [1.31–4.72]	0.003	2.20 [0.86–5.61]	0.099	54 (98.2)	55	0.80 [0.09–7.33]	0.057	NA	NA
Swahili	29 (26.6)	109	1.42 [0.82–2.48]	0.003	1.23 [0.49–3.05]	0.659	105 (96.3)	109	0.12 [0.01–1.12]	0.057	NA	NA
**Understanding of instructions for use**^£^												
Yes	42 (16.4)	256	0.32 [0.18–0.59]	< 0.001	0.44 [0.21–0.91]	0.026	254 (99.2)	256	6.05 [0.98–36.97]	0.063	2.47 [0.31–19.36]	0.391
No	25 (37.9)	66	Reference	-	Reference	-	63 (95.5)	66	Reference	-	Reference	-
**Successful performance**												
Yes	63 (19.9)	317	0.06 [0.01–0.56]	0.007	0.12 [0.01–1.12]	0.063	-	317	-	-	-	-
No	4 (80.0)	5	Reference	-	Reference	-	-	5	-	-	-	-

* P-value calculated using Pearson’s χ_2_ test or Fisher’s exact test;

** P-value calculated using regression analysis;

^μ^ Educational level was categorized according to the educational system of the Democratic Republic of Congo, as follows: (i) low: unschooled or attending primary school; (ii) middle: attending college or technical school; and (iii) high: attending Bachelor’s or graduate degree;

^£^ The correct answers to a minimum of five questions were considered to have understood the instructions for use.

aOR: adjusted odds ratios; cOR: crude odds ratios; CI: confidence interval; NA: not applicable; *P*: *P*-value.

Results of the satisfaction questionnaire concerning the performance of the HIV self-test are shown in S1 Table. Most (96.5%) participants found that the identification of the kit components was easy (74.8% very easy; 21.7% rather easy); 59.6% responded that performing the self-test was very easy, 35.7% found it rather easy, 3.1% found it rather difficult, and 1.6% found it very difficult. When asked about the ability to surmount the difficulties encountered during self-testing, 95.1% of participants found it easy (55% easy; 40.1% rather easy) and only 4, 9% found it difficult (4% rather difficult; 0.9% very difficult).

### Virological performance of HIVST Exacto^®^ Test HIV

**Fig 3:**
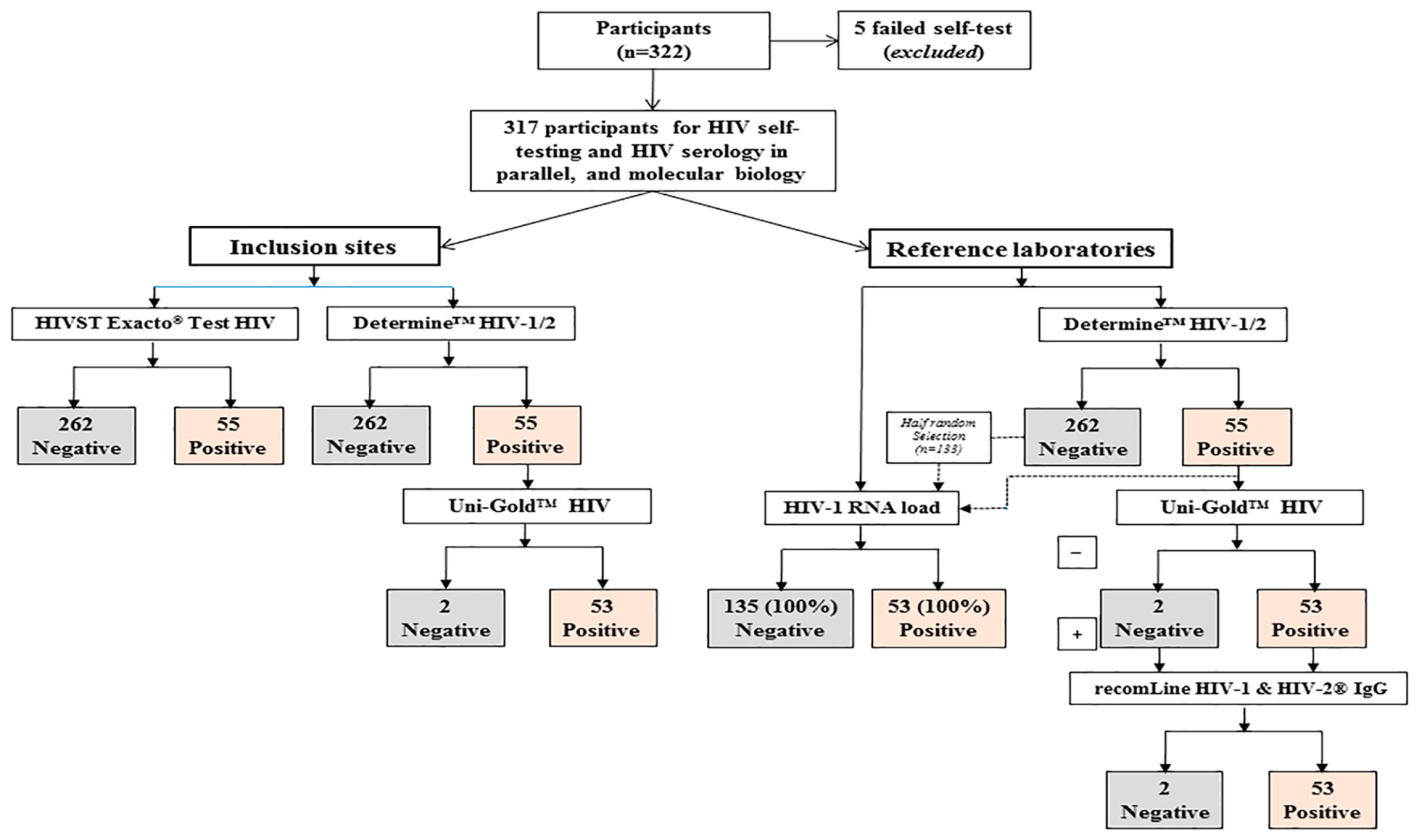
Flow chart showing the results of HIV testing by HIVST Exacto^®^ Test HIV and HIV serology national algorithm in the five sites of inclusion, and by the 2012-revised, 2015-consolidated WHO serological algorithm involving three HIV tests at the Reference Provincial Laboratory of Kisangani and finally by molecular biology (measurement of plasma HIV-1 RNA load) at the reference laboratory of Kinshasa. Among the 322 volunteers, five (1.5%) were unable to collect blood, giving a total of 317 participants to be tested in parallel by the HIVST Exacto^®^ Test HIV, national and 2012-revised, 2015-consolidated WHO serological algorithms and molecular biology. By taking into account the 2012-revised, 2015-consolidated algorithm or the molecular biology for HIV detection as reference diagnosis methods, 262 (82.6%) of the 317 plasmas were non-HIV-infected and 53 (16.7%) were HIV-infected. The HIVST Exacto^®^ Test HIV provided 262 (82.6%) true HIV-negative plasmas and 55 HIV-positive plasmas, including 53 (16.7%) true HIV-positive and two (0.6%) false-positive plasmas.

The results of the virological evaluation of the HIVST Exacto^®^ Test HIV are depicted in Fig 3. Among the 322 volunteers, five (1.5%) were unable to collect blood and were thus unable to provide a valuable result by the HIVST Exacto^®^ Test HIV. A total of 317 participants enrolled in the five sites of inclusion were then tested in parallel by HIVST Exacto^®^ Test HIV and HIV serology national algorithm, and further by the 2012-revised, 2015-consolidated WHO serological algorithm, which involved three HIV tests. Finally, 53 HIV-positive sera, two indeterminate sera, and 133 negative sera obtained after half-random selection of 262 negative sera were analyzed by molecular biology.

The HIVST Exacto^®^ Test HIV provided 262 (82.6%) true HIV-negative plasmas and 55 HIV-positive plasmas, including 53 (16.7%) true HIV-positive and two (0.6%) false-positive plasmas. Thus, the sensitivity of the HIVST Exacto^®^ Test HIV may be estimated at 100% (95% CI; 98.8–100.0), its specificity at 99.2% (95% CI; 97.5–99.8), its PPV in the former Province Orientale at 74.6% (95% CI; 69.5–79.1), and its NPV at 100% (95% CI; 98.8–100.0). The Youden’ J index was calculated at 99.2% (95% CI; 97.5–99.8), demonstrating high efficiency to diagnose HIV infection.

## Discussion

Herein we evaluated the practicability of a prototype immunochromatographic HIV self-tests in Kisangani and Bunia, DRC, according to WHO recommendations [25]. The results of practicability showed that the French instructions for use were used in only one-half of cases, and that the instructions for use in vernacular languages were also frequently preferred, stressing the need for translation and adaptation of the user’s guide in vernacular languages. However, the instructions for use were correctly understood in only 79.5% of the volunteers. The final results were correctly interpreted in 90.2% of cases. Among the positive, negative, and invalid self-tests, misinterpretation occurred in 6.5%, 11.2%, and 16.0% of self-tests, respectively. Finally, HIVST was carried out correctly in 98.4% of cases, although oral assistance was required in 20.8% of cases. In 1.6% of cases, self-testing was difficult or impossible to achieve because of the difficulty in self-blood sampling. In the present series, the lower the educational level of the general public, the greater the difficulty in performing the HIV self-test and interpreting it correctly.

These observations, novel in French-speaking sub-Saharan Africa, on a large number of the general public living in DRC demonstrate: (i) the need to adapt the instructions for use among the Congolese general public, including educational pictograms as well as instructions for use in vernacular language(s) in addition to the French language; (ii) the frequent difficulties in understanding the instructions for use in addition to frequent misinterpretations of test results; and (iii) the generally good practicability of the HIV self-test despite some limitations. Taken together, the study highlights the feasibility of HIVST in French-speaking settings, but the need for an appropriate strategy like supervised HIVST among poorly educated people should be necessary.

### Adaptation of the instructions for use of the Exacto^®^ Test HIV to the Congolese general public

The study had two preliminary prerequisites. The first was to adapt the professional instructions for use of the Exacto^®^ PRO Test HIV into a simplified but comprehensive version for the Congolese general public, with the inclusion of pictures showing African people carrying out the test. The second prerequisite was to be as close as possible to the general adult population, with special attention to those with a low educational level. Indeed, for the HIVST evaluation, the WHO recommends that participants be representative of intended users of the device and include those with different levels of education and socioeconomic status [25]. Specifically, we first took into consideration that a large proportion of the population living in French-speaking sub-Saharan Africa, especially in Central Africa, are uneducated or poorly educated, and thus use local vernacular language(s) instead of the French language [38]. Therefore, the translation of the instructions for use of the HIV self-test in vernacular language(s) appeared to be an important prerequisite for the study. Such an approach could likely become an important step for the implementation of HIVST in sub-Saharan Africa. In practice, the simplified French instructions for use were translated into Lingala and Swahili, which constitute the two most spoken national vernacular languages of the DRC [39].

### Practicability of the Exacto^®^ Test HIV with instructions for use adapted to the Congolese general public

#### Substudy 1

Substudy 1 evaluated the ability of the study participants to understand the instructions for use of the Exacto^®^ Test HIV. The expected results of substudy 1 are of importance for the following practicability substudies and further implementation of HIVST because it is mandatory to check that the instructions for use can be read and understood by all users. Overall, 256 (79.5%) participants who were not previously trained correctly used the finger-stick whole-blood self-test either autonomously or with oral assistance, and thus correctly understood the instructions for use, thus correctly answering at least five questions. Our observations emphasize the need for the translation of the instructions for use in vernacular language(s), and point out that educational level may be one of the limiting factors for practical use of HIV self-tests in a sub-Saharan African cultural context. However, links were observed between the language used for the instructions for use, educational level, and ultimate comprehension of the instructions, which appeared to be associated with educational level and language use. Thus, correct interpretation was significantly less frequent in participants with low or middle educational levels than in those with high educational levels. Furthermore, correct interpretation was less frequent with the instructions for use in Lingala or Swahili than in French. In bivariate analysis, the level of education and the language of the instructions for use were significantly associated with the final understanding. However, after controlling for possible confounders and assessing the independent variable by multivariate logistic regression analysis, the only variable associated with final understanding was the language used. In other terms, vernacular languages were frequently used by poorly and middle-educated people, but the final interpretation was less accurate with vernacular languages than with French, independent of educational level. These observations demonstrate the importance of the translation of the instructions for use into vernacular versions to increase public accessibility to HIVST [22], but also point out the difficulties of such translations in providing easy reading and understanding in poorly educated users. In other terms, although the translation of instructions for use appears important, translation alone might not be sufficient, and other aids such as testing presentation or the use of pictures are likely needed as literacy remains a challenge for some of the target population.

#### Substudy 2

Substudy 2 allowed for the evaluation of the ability of participants to read and interpret the Exacto^®^ Test HIV results. Most participants claimed that interpretation was rather easy or very easy. However, around 10% of test results were misinterpreted. The high frequency of misinterpretation in our study population differed greatly from those reported by Prazuck and colleagues in a non-trained general population living in France in which the vast majority of participants succeeded, with only 2.9% of the participants making errors, mostly when reading an invalid test [10].

#### Substudy 3

Substudy 3 evaluated the ability of participants to use the Exacto^®^ finger-stick whole-blood self-test and get a valid result in a supervised setting. The majority of participants correctly used the self-test and succeeded in obtaining an interpretable result, whereas less than one of 60 participants failed. The general public and health care workers carried out the self-test similarly, and generally autonomously. The correct use of the lancet to collect capillary blood was the most difficult step encountered, and was the first concern raised in oral assistance; persistent incorrect use of the lancet was always associated with HIVST failure. Difficulties in blood collection, although infrequent, might be an inherent limitation of finger-stick whole-blood HIV self-tests, and highlight the potential of oral fluid HIV self-tests which may be preferred in some settings [40].

Around one in five participants needed technical oral assistance and support, especially those using the instructions for use in vernacular languages, as well as those with low and middle educational levels. These features emphasize the importance, and probably the need, to propose a hotline at the time of commercialization of the HIV self-test.

### Analytical performance of the Exacto^®^ Test HIV

The virological performance of the HIV self-test in French-speaking sub-Saharan Africa countries, particularly in Central African countries, is poorly established, while the risk of false-negative and false-positive results in HIV testing is considered to be high in Central Equatorial Africa [41–45]. In the present evaluation, the sensitivity of the Exacto^®^ Test HIV was estimated at 100%, its specificity at 99.2%, its PPV in the former Province Orientale at 74.6%, and its NPV of 100%. The analytical performance of the Exacto^®^ Test HIV used in the DRC, which constitutes an area of circulating HIV-1 strains with broad genetic diversity [46], are thus within the limits required by the WHO (*i*.*e*. sensitivity ≥99.0% and specificity ≥98.0%) [19], making this HIV self-test suitable for routine use in the general adult population in the DRC.

### Study limitations

The study has some limitations: First, it comprised a convenience sample of the general public and health care workers who consented to the evaluation of the practicability of finger-stick whole-blood HIV self-tests, according to WHO recommendations. Selection and volunteer bias are thus probable. Second, the presence of health care observers in self-testing rooms, mimicking telephone support (because of the lack of a hotline on HIV screening and self-testing in the study areas), could constitute a possible limitation in the study design, since the extra effort needed to make a phone call to ask for support in a non-study setting could be a hurdle for some people.

### Conclusions and perspectives

HIVST in French-speaking sub-Saharan Africa countries, particularly in Central African countries, was poorly established. Our field experience with the Exacto^®^ Test HIV self-test demonstrates in the cultural context of Central Africa satisfactory success rates of interpretation performance and its potential for use by the general public with a sufficient educational level. The main obstacle for HIVST was educational level, with execution and interpretation difficulties among poorly educated people.

Two self-testing strategies, supervised and unsupervised, have been documented and evaluated globally [40,47–50]. In an unsupervised self-testing strategy, the self-tester performs the self-test on his or her own without any help, and counselling and linkages are offered either by health care structures or communities or over the phone by trained counsellors or, eventually, through pharmacies [16,51]. Our pilot study generated evidence on the feasibility of the operationalization of a self-testing strategy in participants with a sufficient educational level living in the cultural context of Central Africa. The successful conduct of self-tests may have been related to the use of adapted instructions for use, generally in the French language, provided to sufficiently literate participants who could comprehend them, as well as the fact that by volunteering to participate in the study, these participants showed interest in conducting self-tests by themselves.

Otherwise, our observations also indicate the potential of using a supervised self-testing strategy among poorly educated people, in which testing and counselling processes are aided at all times by a healthcare or nonhealth care professional as a counsellor to read the test results and provide counselling, with a very high rate of acceptability, feasibility, and accuracy in terms of the correct interpretation of test results [40,48]. The evidence of high acceptability for supervised strategies is clear, especially in resource-constrained settings [40]. Furthermore, in a supervised self-testing strategy, HIV self-tests can be provided in bulk by the healthcare or community personnel, thus decreasing the cost of HIVST. For poor and less literate populations who cannot afford HIV self-tests or cannot easily comprehend the process of testing (as in around one in five of this study’s participants), the supervised self-testing strategy may likely remain the best option. However, the supervised option requires a careful, controlled evaluation in many sub-Saharan settings, while the mode and medium of preferences for counselling vary across settings, populations, and strategies. More research on exploring the best strategy (*i*.*e*. supervised *versus* unsupervised strategies) for different high- and low- risk populations in resource-constrained settings is therefore needed.

## Supporting information

S1 Table Items and results of the satisfaction questionnaire concerning the substudy 1, the substudy 2, and the substudy 3. (DOC)

S1 Appendix Full data of substudy 1. (XLSX)

S2 Appendix Study questionnaires in French (original language). (DOC)

S3 Appendix Study questionnaires in English. (DOC)

S4 Appendix Letter of permission from Biosynex to publish Fig 1 in Swahili. (PDF)
