# Supplementary material for: Evaluation of the practicability and virological performance of finger-stick whole-blood HIV self-testing in French-speaking sub-Saharan Africa
Source: PLoS One. 2018 Jan 10;13(1):e0189475. doi: 10.1371/journal.pone.0189475 (PMC5761859; doi:10.1371/journal.pone.0189475)
Supplement: S1 Table — (DOC) [file pone.0189475.s001.doc]

Items and results of the satisfaction questionnaire concerning the instruction notice (substudy 1), the interpretation of HIV self-test results (substudy 2) and the realization of the HIV self-test (substudy 3).

**Satisfaction questionnaire Number (%)**

- **Instruction notice (substudy 1)**
- Information regarding the contents of the kit
  - - - Sufficient 287 (89.1)
      - Insufficient 31 (9.7)
      - Not read 4 (1.2)
- Information concerning the realization of the HIV self-test
- Sufficient 289 (89.6)
- Insufficient 33 (10.2)
- Not read 0 (0.0)
- Information concerning the interpretation of the HIV self-test results
  - - - Sufficient 291 (90.4)
      - Insufficient 30 (9.3)
      - Not read 1 (0.3)
- Overall understanding of the instruction notice
  - - - Very easy 211 (65.5)
      - Rather easy 100 (31.1)
      - Rather difficult 7 (2.2)
      - Very difficult 4 (1.2)
- The use of local language notice
  - Essential 116 (36.0)
  - Useful 159 (49.4)
  - Rather useful 28 (8.7)
  - Unuseful 19 (5.9)
- **Interpretation of HIV self-test results (substudy 2)**
- Reading and interpretation of a ***positive*** test
  - - - Very easy 191 (59.3)
      - Rather easy 111 (34.5)
      - Rather difficult 8 (2.5)
      - Very difficult 12 (3.7)
- Reading and interpretation of a ***negative*** test
  - - - Very easy 209 (64.9)
      - Rather easy 97 (30.1)
      - Rather difficult 8 (2.5)
      - Very difficult 8 (2.5)
- Reading and interpretation of an ***invalid*** test
  - - - Very easy 208 (64.6)
      - Rather easy 96 (29.8)
      - Rather difficult 12 (3.7)
      - Very difficult 6 (1.9)
- Correct observation of blood deposit in the SQUARE well
  - - - Very easy 209 (64.9)
      - Rather easy 101 (31.4)
      - Rather difficult 7 (2.2)
      - Very difficult 4 (1.2)
- **Realization of the HIV self-test (substudy 3)**
- Recognition of the components of the HIV self-test
  - - - Very easy 241 (74.8)
      - Rather easy 70 (21.7)
      - Rather difficult 9 (2.8)
      - Very difficult 2 (0.6)
- Overall realization of the HIV self-test
  - - Very easy 192 (59.6)
    - Rather easy 115 (35.7)
    - Rather difficult 10 (3.1)
    - Very difficult 5 (1.6)
- Ability to surmount the difficulties encountered
  - - Very easy 177 (55.0)
    - Rather easy 129 (40.1)
    - Rather difficult 13 (4.0)
    - Very difficult 3 (0.9)
