# Supplementary material for: Evaluation of the practicability and virological performance of finger-stick whole-blood HIV self-testing in French-speaking sub-Saharan Africa
Source: PLoS One. 2018 Jan 10;13(1):e0189475. doi: 10.1371/journal.pone.0189475 (PMC5761859; doi:10.1371/journal.pone.0189475)
Supplement: S2 Appendix — (DOC) [file pone.0189475.s003.doc]

**Sous-étude 1 - Exacto® Test HIV -**

**COMPREHENSION DE LA NOTICE**

**Questionnaire d’évaluation de la compréhension de la notice**

***A remplir par le participant.***

Date :…..…/………/………….. Site :………………………………. ……….....…………………………

Langue de la notice : Français  Lingala  Swahili

*Au sujet de la notice d’utilisation de l’autotest Exacto® Test HIV (Biosynex, Strasbourg, France):*

**Compréhension des informations liées à la réalisation de l’autotest *Exacto®*:**

1. « *Une lettre en majuscule est associée à chaque composante du kit pour mieux l’identifier à chaque étape de réalisation de l’autotest sur la notice* » :

VRAI  FAUX  Je ne sais pas

1. « *Le prélève-goutte m’aide à recueillir la goute de sang et la déposer immédiatement dans le puits carré de l’autotest* » :

VRAI  FAUX  Je ne sais pas

1. « *Deux gouttes de diluant doivent être déposées au même puits que la goutte de sang* » :

VRAI  FAUX  Je ne sais pas

1. « *Je dois disposer d’un minuteur (montre ou mobile) pour chronométrer les 10 minutes avant de lire le résultat* » :

VRAI  FAUX  Je ne sais pas

**Compréhension des informations liées à l’interprétation du résultat de l’autotest *Exacto®*:**

1. « *Un autotest qui ne présente aucune bande, signifie que le test est négatif* » :

VRAI  FAUX  Je ne sais pas

1. « *Un autotest qui ne présente pas de bande contrôle, signifie que le test est invalide» :*

VRAI  FAUX  Je ne sais pas

1. « *Si mon autotest est* ***positif****, je dois effectuer un second test au laboratoire pour confirmer le résultat de l’autotest* » :

VRAI  FAUX  Je ne sais pas

1. « *J’ai eu des rapports sexuels non-protégés avec une personne inconnue il y a 3 semaines. Avec un autotest Exacto® Test HIV* ***négatif*** *dans cette situation, je suis certain de ne pas avoir été contaminé ?* » :

VRAI  FAUX  Je ne sais pas

**Questionnaire de satisfaction par rapport à la compréhension de la notice de l’autotest *Exacto®*:**

1. *Les informations fournies concernant le contenu du kit vous semblent :*

Suffisantes  Insuffisantes  Je n’ai pas lu ces informations

1. *Les informations fournies concernant la réalisation de cet autotest vous semblent :*

Suffisantes  Insuffisantes  Je n’ai pas lu ces informations

1. *Les informations fournies concernant l’interprétation du résultat vous semblent :*

Suffisantes  Insuffisantes  Je n’ai pas lu ces informations

1. *La compréhension de la notice en général vous semble :*

Très facile  Plutôt facile  Plutôt difficile  Très difficile

1. *La compréhension de la notice en langue locale (lingala ou swahili) vous semble :*

Très facile  Plutôt facile  Plutôt difficile  Très difficile

1. *L’utilisation d’une notice en langue locale (lingala ou swahili) vous semble :*

Indispensable  Utile  Assez utile  Inutile

**Sous-étude 2 - Exacto® Test HIV -**

**GRILLE DE LECTURE DES RESULTATS D’UN PANEL D’AUTOTESTS EXACTO® TEST HIV DEJA PREPARES**

**Interprétation des résultats de l’autotest**

*A compléter par l’observateur.*

*Déroulement de l’étude :*

*1. Vous avez pris connaissance de la grille de lecture de l’autotest Exacto® Test HIV (Biosynex, Strasbourg, France), à tout moment vous pouvez de nouveau la consulter.*

*2. Parmi les 13 autotests Exacto® Test HIV déjà préparés proposés (ou « cassette »), choisissez en un, au hasard.*

*3. Précisez le numéro du test sur cette fiche.*

*4. Demandez et notez le résultat lu par le participant, et notez le résultat attendu.*

5. Répétez les étapes 3 et 4 pour 12 autres tests que vous tirerez au hasard parmi les tests restants.

| **Numéro de la cassette** | **Résultat lu par le volontaire** | | | | **Résultat attendu** | | |
| --- | --- | --- | --- | --- | --- | --- | --- |
|  | **POSITIF** | **NEGATIF** | **INVALIDE** | **NE SAIT PAS** | **Positif** | **Négatif** | **Invalide** |
|  |  |  |  |  |  |  |  |
|  |  |  |  |  |  |  |  |
|  |  |  |  |  |  |  |  |
|  |  |  |  |  |  |  |  |
|  |  |  |  |  |  |  |  |
|  |  |  |  |  |  |  |  |
|  |  |  |  |  |  |  |  |
|  |  |  |  |  |  |  |  |
|  |  |  |  |  |  |  |  |
|  |  |  |  |  |  |  |  |
|  |  |  |  |  |  |  |  |
|  |  |  |  |  |  |  |  |
|  |  |  |  |  |  |  |  |

**Sous-étude 3 - Exacto® Test HIV -**

**manipulation de l’autotest par un utilisateur PROFANE**

**Observation de la manipulation de l’autotest par un utilisateur profane**

*A compléter par l’observateur.*

*L’observateur met à la disposition du participant une boite l’autotest Exacto® Test HIV (Biosynex, Strasbourg, France) avec une notice dont la langue (français, lingala ou swahili) est choisie par le participant. Il explique au participant son rôle et explique qu’il joue le rôle de l’assistance téléphonique à tout moment pendant la manipulation du test, si le participant en fait la demande.*

| Nom et prénom de l’observateur : ……………………………………………………..  Langue de la notice : Français  Lingala  Swahili | | DATE :  ……../……../…………. |
| --- | --- | --- |
| Items |  | Observation |
|  | *Heure de début d’observation* |
| **#1** | **Le participant a-t-il reconnu les différentes composantes du kit ?** | **OUI**  **/ NON** |
| **#2** | **S’est-il lavé les mains ?** | **OUI**  **/ NON** |
| **#3** | **A-t-il trouvé la cassette dans le sachet ?** | **OUI**  **/ NON** |
| **#4** | **A-t-il ouvert le flacon de diluant ?** | **OUI**  **/ NON** |
| **#5** | **S’est-il correctement désinfecté le doigt ?** | **OUI**  **/ NON** |
| **#6** | **A-t-il essuyé les traces d’alcool avec la compresse ?** | **OUI**  **/ NON** |
| **#7** | **A-t-il utilisé correctement l’autopiqueur ?** | **OUI**  **/ NON** |
| **#8** | **A-t-il formé une grosse goutte de sang ?** | **OUI**  **/ NON** |
| **#9** | **A-t-il su utiliser le prélève-goutte ?** | **OUI**  **/ NON** |
| **#10** | **A-t-il vérifié que le prélève-goutte était rempli de sang?** | **OUI**  **/ NON** |
| **#11** | **A-t-il déposé le sang dans le puits carré SANG ?** | **OUI**  **/ NON** |
| **#12** | **A-t-il déposé deux gouttes de diluant dans le puits rond DILUANT ?** | **OUI**  **/ NON** |
| **#13** | **A-t-il enclenché un chronomètre (ou équivalent) ?** | **OUI**  **/ NON** |
|  | *Heure de fin de manipulation* |  |
|  | Le participant a-t-il demandé un soutien oral (appel téléphonique) durant le temps de la manipulation ? | **OUI**  **/ NON** |
| Si **OUI** à quelle(s) étape(s) (numéro de l’item) :  **Question(s) : Réponse(s) :** | | |

*Pour finir cette étude, le participant doit remplir un questionnaire satisfaction.*

**Sous-étude 4 - Exacto® Test HIV -**

**UTILISATION DE L’AUTOTEST : QUESTIONNAIRE DE SATISFACTION**

**Satisfaction concernant la réalisation et la lecture de l’autotest**

*A compléter par le participant.*

Profane  Professionnel de la santé

Langue de la notice : Français  Lingala  Swahili

**Votre profil :**

- **Age :** …………….. ans
- **Origine géographie (ville de résidence)** **:** Kisangani  Bunia  Autres………….................
- **Sexe :** Femme  Homme
- **Si femme, êtes-vous enceinte avec test de grossesse positif ?** Oui  Non
- **Etat civil :** Célibataire  Mariage ou union libre  Veuf(ve)  Divorcé(e) ou séparé(e)
- **Occupation :** Elève/Etudiant  Avec emploi  Sans emploi
- **Niveau d’étude :** Non scolarisé  Primaire  Secondaire

Supérieur ou universitaire : 1^er^ cycle  2^e^ cycle  3^e^ cycle

- **Nombre de partenaires sexuel(le)s depuis 6 mois:** Aucun  1 à 5  6 à10  ≥10
- **Relations sexuelles depuis 6 mois avec** :  Hommes  Femmes  Les deux
- **Risque d’exposition** (rapport sexuel non-protégé, contact sanguin ou blessure par un objet tranchant contaminé, etc.) **depuis 6 mois :** Oui  Non
- **Si oui, le(s)quel(s) :**…………………………………………………………………………………….
- **Avez-vous déjà effectué un test de dépistage VIH** **:** Oui  Non
- **Avez-vous déjà effectué un test de grossesse (***si femme***):** Oui  Non
- **Avez-vous déjà effectué un autre test rapide (***glycémie, etc***):** Oui  Non
- **Si oui, le(s)quel(s) :**…………………………………………………………………………………….
- **Savez-vous si vous êtes infectés par le VIH** **:** Oui  Non
- **Si oui à la question précédente, prenez-vous un traitement antirétroviral ?** Oui  Non

**Au sujet de la manipulation pratique**

**de l’autotest Exacto® Test HIV (Biosynex, Strasbourg, France):**

1. *Comment évaluez-vous l’identification des composants du kit de l’autotest sur la notice :*

Très facile  Plutôt facile  Plutôt difficile  Très difficile

1. *Comment évaluez-vous la réalisation de l’autotest Exacto® Test HIV :*

Très facile  Plutôt facile  Plutôt difficile  Très difficile

1. *Comment évaluez-vous l’utilisation de l’auto-piqueur :*

Très facile  Plutôt facile  Plutôt difficile  Très difficile

1. *Comment évaluez-vous le recueil de la goutte de sang par le prélève-goutte :*

Très facile  Plutôt facile  Plutôt difficile  Très difficile

1. *Comment évaluez-vous la* ***reconnaissance*** *de chaque* ***puits*** *de la cassette test où sont déposées la goutte de sang et les gouttes de diluant :*

Très facile  Plutôt facile  Plutôt difficile  Très difficile

1. *Comment évaluez-vous la manière dont vous avez surmonté les difficultés rencontrées éventuelles :*

Très facile  Plutôt facile  Plutôt difficile  Très difficile

**Au sujet de la lecture du résultat de l’autotest *Exacto®*:**

1. *Comment évaluez-vous la lecture des* ***bandes contrôle C*** *et* ***test T*** *sur l’autotest* :

Très facile  Plutôt facile  Plutôt difficile  Très difficile

1. *Comment évaluez-vous l’interprétation du* ***résultat positif*** *de l’autotest*:

Très facile  Plutôt facile  Plutôt difficile  Très difficile

1. *Comment évaluez-vous l’interprétation du* ***résultat négatif*** *de l’autotest*:

Très facile  Plutôt facile  Plutôt difficile  Très difficile

1. *Comment évaluez-vous l’interprétation du* ***résultat invalide ou ininterprétable*** *de l’autotest*:

Très facile  Plutôt facile  Plutôt difficile  Très difficile

1. *Comment avez-vous observé le dépôt de sang visible dans le puits carré SANG après avoir effectué l’autotest*:

Très facile  Plutôt facile  Plutôt difficile  Très difficile

| **Commentaire libre sur l’autotest Exacto®:**  **……………………………………………………………………………………………………………………………….**  **……………………………………………………………………………………………………………………………….**  **……………………………………………………………………………………………………………………………….**  **……………………………………………………………………………………………………………………………….**  **……………………………………………………………………………………………………………………………….**  **……………………………………………………………………………………………………………………………….**  **……………………………………………………………………………………………………………………………….**  **……………………………………………………………………………………………………………………………….**  **……………………………………………………………………………………………………………………………….**  **……………………………………………………………………………………………………………………………….**  **……………………………………………………………………………………………………………………………….**  **……………………………………………………………………………………………………………………………….** |
| --- |

**Sous-étude 5 - Exacto® Test HIV -**

**VALIDATION DES PERFORMANCES CLINIQUES SUR SITE**

*A compléter par le participant, l’observateur et l’operateur*

Date : ………/………/….………. ; Site :……………………………..

Langue de la notice : Français  Lingala  Swahili

**Résultat de l’autotest Exacto® Test HIV**

**(Biosynex, Strasbourg, France)**

**Participant**

**Bande contrôle C**: Absence  Présence  Douteux

**Bande test T**: Absence  Présence  Douteux

**Dépôt de sang visible**

**dans le puits carré SANG** : Absence  Présence  Douteux

**Résultat de l’autotest :** Négatif  Positif  Invalide

**Observateur**

**Bande contrôle C**: Absence  Présence  Douteux

**Bande test T**: Absence  Présence  Douteux

**Dépôt de sang visible**

**dans le puits carré SANG** : Absence  Présence  Douteux

**Résultat de l’autotest :** Négatif  Positif  Invalide

Nom de l’observateur:…………………………………………….

**Test de dépistage de l’infection à VIH réalisé sur site**

**Operateur**

**🡺Utilisation de tests rapide d’orientation diagnostique (TROD) en parallèle:**

- **Determine® :** Négatif  Positif  Douteux
- **Unigold® :** Négatif  Positif  Douteux
- **RecomLine® :** Négatif  Positif  Douteux

**🡺Interprétation**

- **Selon l’algorithme II OMS 1997 :** Négatif  Positif  Inconclusif
- **Selon l’algorithme tie-breaker OMS 2012 :**

Négatif  Positif  Inconclusif

Nom de l’Operateur : ……………………………………………...
