# Supplementary material for: Evaluation of the practicability and virological performance of finger-stick whole-blood HIV self-testing in French-speaking sub-Saharan Africa
Source: PLoS One. 2018 Jan 10;13(1):e0189475. doi: 10.1371/journal.pone.0189475 (PMC5761859; doi:10.1371/journal.pone.0189475)
Supplement: S3 Appendix — (DOC) [file pone.0189475.s004.DOC]

**Substudy 1 - Exacto® HIV Test -**

**UNDERSTANDING OF THE INSTRUCTIONS**

**Questionnaire to assess understanding of the instructions**

***To be completed by the participant.***

Date:…..…/………/………….. Site:………………………………. ……….....…………………………

Language of the instructions: French  Lingala  Swahili

*Concerning the instructions for use of the Exacto® HIV Self-Test (Biosynex, Strasbourg, France):*

**Understanding of the information related to doing the *Exacto®* self-test:**

1. *“In the instructions, a capital letter is associated with each of the kit components so that they be more easily identified at each stage of doing the self-test”:*

TRUE  FALSE  I don't know

1. *“The sampler stick helps me to collect the drop of blood and to deposit it into the self-test's square well straight away”:*

TRUE  FALSE  I don't know

1. *“Two drops of diluent must be deposited into the same well as the drop of blood”:*

TRUE  FALSE  I don't know

1. *“I must have a timer (watch or mobile) available to time the 10 minutes before reading the result”:*

TRUE  FALSE  I don't know

**Understanding of the information related to interpreting the result of the *Exacto®* self-test:**

1. *“A self-test that shows no bands means that the test is* ***negative****”:*

TRUE  FALSE  I don't know

1. *“A self-test that shows no control band means that the test is* ***invalid****”:*

TRUE  FALSE  I don't know

1. “*If my self-test is* ***positive****, I have to do a second test in the laboratory to confirm the result of the self-test*”:

TRUE  FALSE  I don't know

1. “*I had unprotected intercourse with a stranger 3 weeks ago. With a* ***negative*** *Exacto® HIV Self-Test in this situation, am I certain that I haven't been infected?*”:

TRUE  FALSE  I don't know

**Satisfaction questionnaire in relation to understanding of the instructions for the *Exacto®* self-test:**

1. *In your opinion, the information provided about the contents of the kit is:*

Adequate  Inadequate  I did not read this information

1. *In your opinion, the information provided about doing this self-test is:*

Adequate  Inadequate  I did not read this information

1. *In your opinion, the information provided about interpreting the result is:*

Adequate  Inadequate  I did not read this information

1. *In your opinion, understanding the instructions in general is:*

Very easy  Rather easy  Rather difficult  Very difficult

1. *In your opinion, understanding the instructions in the local language (Lingala or Swahili) is:*

Very easy  Rather easy  Rather difficult  Very difficult

1. *In your opinion, the use of instructions in the local language (Lingala or Swahili) is:*

Indispensable  Useful  Quite useful  Useless

**Substudy 2 - Exacto® HIV Test -**

**READING GRID FOR THE RESULTS OF PRE-PREPARED SAMPLES OF EXACTO® HIV SELF-TESTS**

**Interpretation of the results of the self-test**

*To be completed by the observer.*

*Running the study:*

*1. You have read the reading grid for the Exacto® HIV Self-Test (Biosynex, Strasbourg, France). You can consult it again at any time.*

*2. From the 13 pre-prepared Exacto® HIV Self-Tests proposed (or “cassette”), choose one at random.*

*3. Write down the number of the test on this form.*

*4. Ask for and write down the result read by the participant, and write down the expected result.*

5. Repeat steps 3 and 4 for 12 other tests that you will choose at random from the remaining tests.

| **Cassette number** | **Result read by the volunteer** | | | | **Expected result** | | |
| --- | --- | --- | --- | --- | --- | --- | --- |
|  | **POSITIVE** | **NEGATIVE** | **INVALID** | **DOESN'T KNOW** | **Positive** | **Negative** | **Invalid** |
|  |  |  |  |  |  |  |  |
|  |  |  |  |  |  |  |  |
|  |  |  |  |  |  |  |  |
|  |  |  |  |  |  |  |  |
|  |  |  |  |  |  |  |  |
|  |  |  |  |  |  |  |  |
|  |  |  |  |  |  |  |  |
|  |  |  |  |  |  |  |  |
|  |  |  |  |  |  |  |  |
|  |  |  |  |  |  |  |  |
|  |  |  |  |  |  |  |  |
|  |  |  |  |  |  |  |  |
|  |  |  |  |  |  |  |  |

**Substudy 3 - Exacto® HIV Test -**

**handling of the self-test by a LAY user**

**Observation of the handling of the self-test by a lay user**

*To be completed by the observer.*

*The observer gives the participant an Exacto® HIV Self-Test (Biosynex, Strasbourg, France) box with instructions, the language of which (French, Lingala or Swahili) is chosen by the participant. The observer explains his/her role to the participant, and explains that he/she will play the role of a helpline agent at any time during the handling of the test if the participant requests it.*

| Surname and given name of the observer: ……………………………………………………..  Language of the instructions: French  Lingala  Swahili | | DATE:  ……../……../…………. |
| --- | --- | --- |
| Items |  | Observation |
|  | *Start time of observation* |
| **1.** | **Did the participant recognise the various kit components?** | **YES**  **/ NO** |
| **2.** | **Did he/she wash his/her hands?** | **YES**  **/ NO** |
| **3.** | **Did he/she find the cassette in the sachet?** | **YES**  **/ NO** |
| **4.** | **Did he/she open the diluent vial?** | **YES**  **/ NO** |
| **5.** | **Did he/she disinfect his/her finger correctly?** | **YES**  **/ NO** |
| **6.** | **Did he/she wipe away the traces of alcohol with the compress?** | **YES**  **/ NO** |
| **7.** | **Did he/she use the lancet correctly?** | **YES**  **/ NO** |
| **8.** | **Did he/she form a large drop of blood?** | **YES**  **/ NO** |
| **9.** | **Was he/she able to use the pipette?** | **YES**  **/ NO** |
| **10.** | **Did he/she check that the pipette was filled with blood?** | **YES**  **/ NO** |
| **11.** | **Did he/she deposit the blood into the BLOOD square well?** | **YES**  **/ NO** |
| **12.** | **Did he/she deposit two drops of diluent into the DILUENT round well?** | **YES**  **/ NO** |
| **13.** | **Did he/she start a timer (or equivalent)?** | **YES**  **/ NO** |
|  | *End time of handling* |  |
|  | Did the participant ask for verbal support (telephone call) during the handling? | **YES**  **/ NO** |
| If **YES** at what stage(s) (item number):  **Question(s): Response(s):** | | |

*To finish this study, the participant must complete a satisfaction questionnaire.*

**Substudy 4 - Exacto® HIV Test -**

**USE OF THE SELF-TEST: SATISFACTION QUESTIONNAIRE**

**Satisfaction concerning the administration and reading of the self-test**

*To be completed by the participant.*

Layperson  Healthcare professional

Language of the instructions: French  Lingala  Swahili

**Your profile:**

- **Age:** …………….. years
- **Geographical origin (city of residence):** Kisangani  Bunia  Other………….................
- **Sex:** Female  Male
- **If you are a woman, are you pregnant with a positive pregnancy test?** Yes  No
- **Marital status:** Single  Married or cohabiting  Widowed  Divorced or separated
- **Occupation:** Student  Employed  Unemployed
- **Level of education:** Unschooled  Primary  Secondary

Higher or university: Bachelor  Master  Doctorate

- **Number of sexual partners in the last 6 months:** None  1 to 5  6 to 10  ≥ 10
- **Intercourse in the last 6 months with:**  Men  Women  Both
- **Risk of exposure** (unprotected intercourse, blood contact or injury from a contaminated sharp object, etc.) **in the last 6 months:** Yes  No
- **If yes, which one(s):**…………………………………………………………………………………….
- **Have you ever done an HIV screening test:** Yes  No
- **Have you ever done a pregnancy test (***if woman***):** Yes  No
- **Have you ever done another rapid test (***blood sugar, etc*.**):** Yes  No
- **If yes, which one(s):**…………………………………………………………………………………….
- **Do you know whether you are infected with HIV:** Yes  No
- **If you answered yes to the previous question, are you taking an antiretroviral treatment?** Yes  No

**In terms of the practical handling**

**of the Exacto® HIV Self-Test (Biosynex, Strasbourg, France):**

1. *How easy or difficult was it to identify the components of the self-test kit in the instructions:*

Very easy  Rather easy  Rather difficult  Very difficult

1. *How easy or difficult was it to do the Exacto® HIV Self-Test:*

Very easy  Rather easy  Rather difficult  Very difficult

1. *How easy or difficult was it to use the lancet?*

Very easy  Rather easy  Rather difficult  Very difficult

1. *How easy or difficult was it to collect the drop of blood with sampler stick:*

Very easy  Rather easy  Rather difficult  Very difficult

1. *How easy or difficult was it to* ***recognise*** *each* ***well*** *in the cassette test where the drop of blood and the drops of diluent are deposited:*

Very easy  Rather easy  Rather difficult  Very difficult

1. *How easy or difficult was it to overcome any possible difficulties faced:*

Very easy  Rather easy  Rather difficult  Very difficult

**In terms of the reading of the *Exacto®* self-test result:**

1. *How easy or difficult was it to read the* ***C control*** *and* ***T test bands*** *on the self-test*:

Very easy  Rather easy  Rather difficult  Very difficult

1. *How easy or difficult was it to interpret the* ***positive result*** *of the self-test*:

Very easy  Rather easy  Rather difficult  Very difficult

1. *How easy or difficult was it to interpret the* ***negative result*** *of the self-test*:

Very easy  Rather easy  Rather difficult  Very difficult

1. *How easy or difficult was it to interpret the* ***invalid or uninterpretable result*** *of the self-test*:

Very easy  Rather easy  Rather difficult  Very difficult

1. *How easy or difficult did you find it to observe the visible deposit of blood in the BLOOD square well after doing the self-test*:

Very easy  Rather easy  Rather difficult  Very difficult

| **Open-ended comments about the Exacto® self-test:**  **……………………………………………………………………………………………………………………………….**  **……………………………………………………………………………………………………………………………….**  **……………………………………………………………………………………………………………………………….**  **……………………………………………………………………………………………………………………………….**  **……………………………………………………………………………………………………………………………….**  **……………………………………………………………………………………………………………………………….**  **……………………………………………………………………………………………………………………………….**  **……………………………………………………………………………………………………………………………….**  **……………………………………………………………………………………………………………………………….**  **……………………………………………………………………………………………………………………………….**  **……………………………………………………………………………………………………………………………….**  **……………………………………………………………………………………………………………………………….** |
| --- |

**Substudy 5 - Exacto® HIV Test -**

**VALIDATION OF ONSITE CLINICAL PERFORMANCE**

*To be completed by the participant, the observer and the operator*

Date: ………/………/….………. ; Site :……………………………..

Language of the instructions: French  Lingala  Swahili

**Result of the Exacto® HIV Self-Test**

**(Biosynex, Strasbourg, France)**

**Participant**

**C control band**: Absent  Present  Uncertain

**T test band**: Absent  Present  Uncertain

**Visible deposit of blood**

**in the BLOOD square well**: Absent  Present  Uncertain

**Result of the self-test:** Negative  Positive  Invalid

**Observer**

**C control band**: Absent  Present  Uncertain

**T test strip**: Absent  Present  Uncertain

**Visible deposit of blood**

**in the BLOOD square well**: Absent  Present  Uncertain

**Result of the self-test:** Negative  Positive  Invalid

Name of the observer:…………………………………………….

**Screening test for HIV infection performed on site**

**Operator**

**🡺Use of rapid diagnostic tests (RDTs) at the same time:**

- **Determine®:** Negative  Positive  Uncertain
- **Unigold®:** Negative  Positive  Uncertain
- **RecomLine®:** Negative  Positive  Uncertain

**🡺Interpretation**

- **Based on the 1997 WHO algorithm II:** Negative  Positive  Inconclusive
- **Based on the 2012 WHO tie-breaker algorithm:**

Negative  Positive  Inconclusive

Name of the Operator: ……………………………………………...
