# Supplementary material for: Evaluation of the practicability and virological performance of finger-stick whole-blood HIV self-testing in French-speaking sub-Saharan Africa
Source: PLoS One. 2018 Jan 10;13(1):e0189475. doi: 10.1371/journal.pone.0189475 (PMC5761859; doi:10.1371/journal.pone.0189475)
Supplement: S4 Appendix — (PDF) [file pone.0189475.s005.pdf]

BIOSYNEX
Easy Diagnostics for Life

Ref. Evaluation of the practicability and virological performances of finger-stick whole blood HIV self-test in French-speaking sub-Saharan Africa

Dear Dr Tonen,

Following your request, I confirm that Biosynex gives permission for the open-access journal PLOS ONE to publish Figure 1 in Swahili featuring Biosynex copyright under the Creative Commons Attribution License (CCAL) CC BY 4.0 (http://creativecommons.org/licenses/by/4.0/). Please be aware that this license allows unrestricted use and distribution, even commercially, by third parties.

We remain at your disposal for any further information.

Best regards

Made in Strasbourg, on June 30th, 2017

Thierry Paper
Deputy General Manager

BIOSYNEX S.A.
12 rue Ettore Bugatti - CS28006
67038 Strasbourg Cedex FRANCE
Tél : +33 3 88 77 57 00 - Fax : +33 3 59 81 21 74
Siret : 481 075 703 00019 - N° CEE : FR11481075703

Biosynex S.A. – 12 rue Ettore Bugatti Eckbolsheim – CS 28006 - 67038 Strasbourg Cedex
Tél : +33 (0)3 88 77 57 21 – Fax : +33 (0)3 59 81 21 74
SA au capital de 805 028 € - RCS : 481 075 703 – APE 2120Z - Lieu de juridiction : Strasbourg
